# Comparison of Milk Odd- and Branched-Chain Fatty Acids among Human, Dairy Species and Artificial Substitutes

**DOI:** 10.3390/foods11244118

**Published:** 2022-12-19

**Authors:** Silvia Carta, Fabio Correddu, Gianni Battacone, Giuseppe Pulina, Anna Nudda

**Affiliations:** Dipartimento di Agraria, University of Sassari, Viale Italia, 39, 07100 Sassari, Italy

**Keywords:** OBCFA, ruminant milk, human milk, infant formula, donkey milk, health

## Abstract

The aim of the study was to compare odd and branched-chain fatty acids (OBCFA) of milk from sheep, goat, cow, buffalo, donkey, human, and formula milk. Ruminant, monogastric, and human milks have different concentrations of these fatty acids (FA). To highlight the differences on OBCFA, a total of 282 individual milk samples were analyzed by gas chromatography. The OBCFA were found higher in ruminant than non-ruminant milks (*p* < 0.05). Among ruminants, sheep milk had the highest OBCFA (4.5 g/100 g of total FAME), whereases the lowest values were found in formula milk (0.18 g/100 g of total FAME). Regarding individual linear odd-chain FA (linear-OCFA), C11:0 was found higher in donkey milk than others, while sheep and buffalo milks had the greatest concentration of C15:0. Among BCFA, the iso-BCFA were higher than anteiso-BCFA in all considered milks. The isoC17:0 showed the highest concentration in all milks except for donkey and buffalo, which showed higher concentration of isoC16:0 than others. In conclusion, ruminant milks are different in terms of these FA compared to human milk and its substitutes. However, the greatest differences were found with formula milk, suggesting that this product needs the implementation of these FA to be more similar to human milk composition.

## 1. Introduction

Milk is considered a complete food, especially for infants, due to its content of essential compounds (amino acids, fatty acids, vitamins, minerals, etc.). Milk and derived foods are also defined nutraceutical foods, because of the presence of bioactive complexes with healthy effects. Extensive literature dealt with the benefits of bioactive peptides [1,2] and peculiar fatty acids [3]. Regarding fatty acids (FA), in the last decades, great attention has been dedicated to polyunsaturated FA of the omega3 family (PUFA n-3) and conjugated linoleic acid (CLA) concentrations of dairy products. More recently, other milk FA, the odd- and branched-chain FA (OBCFA) have attracted increasing interest among scientists. The principal OBCFA in the milk of ruminants are isomers of tetradecanoic acids (isoC14:0), pentadecanoic acid (C15:0, iso and anteisoC15:0), hexadecenoic acid (isoC16:0), and heptadecanoic acid (C17:0, iso and anteisoC17:0). The OBCFA arise mainly as residuals of the ruminal bacterial membranes [4] and, for this reason, are distinctive of ruminant products [5]. Milk and meat from these species represent the main source of OBCFA for humans [6,7], whereas they are found in low amounts in the products from monogastrics [7]. The OBCFA can be found also in human milk, as a consequence of the intake of dairy products and ruminant meat [8]. Endogenous synthesis of OBCFA in humans cannot be excluded, even if very little is known about de novo OBCFA synthesis in internal tissues (e.g., the mammary gland) [9]. The concentration and the proportion among OBCFA can vary depending on different factors, but the animal diet is the principal [5]. In ruminants, a decrease of the forage to concentrate ratio [6,10] and the supplementation of polyphenols [11,12] could lead to a decrease of OBCFA. Moreover, the negative energy balance causing lipid mobilization of body fat and the FAs metabolism in mammary glands can affect the OBCFA content in milk [5]. The positive relationship between OBCFA and human health has been evidenced by several studies highlighting a beneficial role in gut health [5,13] and cardiovascular diseases [14], inflammation [15] and cancer [16]. In particular, the increasing interest on these FA is related to their anticancer activity, evidenced through the inhibition of fatty acids synthesis in tumor cells [17]. OBCFA are also associated to a lower risk of heart diseases [15] and metabolic diseases [18]. Among the OBCFA, C15:0 and C17:0 are known for their positive effects on cardiovascular disease [19], on the incidence of type 2 diabetes [20] and on inflammation and fibrosis [15]. The role of OBCFA in weight loss and weight maintenance has been reported [21]. Recently, it has been evidenced that iso- branched-chain fatty acids (iso-BCFA) could downregulate the expression of genes implicated in lipid synthesis and of genes that codify proinflammatory proteins [22].

The OBCFA could be considered as biomarkers of the rumen function of animals. Some studies showed that cellulosolytic bacteria are rich in iso-BCFA, whereas the linear odd-chain fatty acid (linear-OCFA) and anteiso-BCFA are mainly abundant in amylolytic ones [4,23]. Considering the strong relationship between diet composition and bacteria microflora, OBCFA could also give information about proper rumen function. Moreover, some BCFA might be used as biomarkers to identify diarrheic diseases in animals and humans. For example, the concentrations of total even-chain-branched FA and some individuals as isoC16:0, isoC17:0, anteisoC17:0 were increased in the feces of unhealthy animals [13]. The increase of these FA could be associated with the increase in some branched-chain aminoacids (such as valine, leucine and isoleucine) in animals with diarrhea that could be transformed to α ketoacids to produce isoC16:0, isoC17:0, and anteiso-C17:0 [13]. However, isoC14:0, isoC16:0, isoC18:0, anteisoC15:0, and anteisoC17:0 have demonstrated to have anti-inflammatory functions in intestinal epithelial cell lines [24].

The C15:0 and C17:0 have different origins, and, for this reason, their concentration may vary based on several factors, such as species and diet. Jenkins et al. [19] evidenced a C15:0 to C17:0 ratio approximately equal to 1:2 in human plasma, whereases the same ratio is found about 2:1 in dairy products. In ruminants, these FA are derived mainly from ruminal bacteria cell walls [10], even if it is known that the mammary gland can synthetize them from propionate [25]. Liu et al. [26] suggested that C17:0 might be synthetized partially endogenously due to the higher content of this FA in milk than in duodenal flow. In human plasma, the concentration of C15:0 is strongly related to the intake of dairy products, whereas, for the C17:0 a potential endogenous synthesis has been suggested.

Infant formula is considered substitute for infant feeding, and it is formulated to be as much as possible similar to human breast milk, by using cow milk and/or soy as the base, with the addition of a number of supplemental ingredients of different origin [27]. Donkey milk has become popular for human consumption because it is considered a substitute for human milk, in particular for infants with an intolerance for cow’s milk [28,29]. The FA composition of these types of milk can differ greatly and a huge difference could be observed in the content of beneficial FA, including OBCFA.

The objective of this experimental work was to compare the OBCFA spectrum in human milk and artificial infant formula compared to that of dairy ruminant species (cows, buffalo, goat and sheep), and donkeys.

## 2. Materials and Methods

The number of individual milk samples used for this study were 11 from humans (donated by volunteer mothers), 70 from cows, 61 from buffalo, 69 from goats, 55 from sheep, and 8 from donkeys. The samples used come from our abundant collection of milk of different species obtained in various experimental surveys conducted in Sardinia, except for the buffalo, whose milk comes from Campania, and human, whose milk was voluntarily donated. All samples were frozen immediately after collection and stored at −20 °C. Eight different formula substitutes were purchased at random from local supermarkets.

Fat extraction and FAME preparation were performed as described by Nudda et al. [30]. Analyses of milk OBCFA were carried out by a gas chromatographic method as previously described [31]. Briefly, after the lipid extraction, the FIL-IDF standard procedure [32] was followed for the esterification of FA. Obtained fatty acids methyl esters (FAME) were separated, using a GC FID system equipped with a capillary column (CP-Sil 88 100 × 0.25 × 0.2, Agilent Technologies, Santa Clara, CA, USA), and identified by comparing their retention time with that of a series of analytical standards and consulting previous studies [33,34]. Analytical standard included the Supelco 37 component FAME MIX (Supelco, Bellefonte, PA, USA), the GLC-110 MIX (Matreya Inc. Pleasant Gap, PA, USA) and some individual BCFA (Matreya Inc. Pleasant Gap, PA, USA). Methyl valerate and methyl tridecanoate (Sigma-Aldrich Inc., St. Louis, MO, USA) were used as internal standards. The FA concentration was reported as g/100 g of total FAME.

Data of milk fat concentration and milk OBCFA composition were processed using ANOVA based on the general linear model procedure (PROC GLM) of the software SAS [35], where the type of milk was considered as fixed factor. The Tukey test was used as the post hoc test. Means were declared statistically different when *p* < 0.05.

To obtain an overview of the differences of milk OBCFA profile among milks, a principal component analysis (PCA) was applied on all the considered FA (eleven) of milk using Minitab 17 software (Minitab17). A total 11 principal components were extracted, and individual PC scores and loadings were retained to produce plots of the two first PC.

## 3. Results

### 3.1. Univariate Analysis

Table 1 reports the differences in fat and individual OBCFA concentrations among milks of different species and milk substitutes.

The highest fat concentration was found in buffalo milk, followed by sheep and goats and then by cows. Donkey and human milks exhibited lower fat concentration than ruminant milks (*p* < 0.05). Formula milk had lower fat concentration than buffalo and sheep milks (*p* < 0.05).

In total, 11 FAs were identified: 3 linear-OCFA (C11:0, C:15:0 and C17:0), 5 iso-BCFA (isoC13:0, isoC14:0, isoC15:0, isoC16:0 and isoC17:0) and 3 anteiso-BCFA (anteisoC13:0, anteisoC15:0 and anteisoC17:0). All the considered FA were identified in buffalo, donkey, goat, and sheep milks, whereases anteisoC13:0 in cow milk and isoC13:0 and anteisoC13:0 in human and in formula milk were not detected. The OBCFA were higher in ruminant than monogastric species. Sheep milk displayed higher concentration (4.5 g/100 g of total FAME, *p* < 0.001) of OBCFA than the milk of the other ruminants (*p* < 0.05), mainly due to the high concentration of C15:0 and C17:0 (in all their forms: odd, iso and anteiso), followed by milk of buffaloes, which had higher OBCFA than cows and goats’ milks (*p* < 0.05). Among non-ruminant milks, the lowest value of OBCFA was found in formula milk (about 0.2 g/100 g of total FA), which was about the 10% compared to the concentration of these FA in human milk (1.3 g/100 g of total FAME).

Regarding the linear-OCFA, milk of cow, buffalo, goat, and donkey had similar concentration (mean of 1.66 g/100 g of total FAME), that was lower than that of sheep (*p* < 0.05) and higher compared to that of human and formula milks (*p* < 0.05). The C11:0 concentration was higher in donkey milk samples (0.895 g/100 g of total FAME) compared to the other milks (*p* < 0.05); among ruminants, the highest concentration was found in sheep, followed by goat, cow, and buffalo milk. The C15:0 exhibited higher concentration in sheep and buffalo (mean of 1.16 g/100 g of total FA) than in the cow and goat milks (*p* < 0.05), with cow milk having higher concentration than goat milk (*p* < 0.05). Donkey and human milk had similar concentration of C15:0, a result lower than that of ruminant milks (*p* < 0.05) and higher than that of formula milk (*p* < 0.05). The C17:0 showed higher values in sheep and goat milks (mean of 0.77 g/100 g of total FAME) compared to the buffalo and cow milk (*p* < 0.05), with the first showing higher values (*p* < 0.05). Donkey and human milk had similar concentration of C17:0, a result lower than that of ruminant milks (*p* < 0.05) and higher than that of formula milk (*p* < 0.05).

The highest iso-BCFA, in almost all species, was the isoC17:0, except for donkey and buffalo milk, which had the isoC16:0 as the highest compared to others. Sheep milk had higher concentration of this FA compared to the other milks (*p* < 0.05), goat milk had higher concentration than cow and buffalo milks (*p* < 0.05), which did not differ among them (*p* > 0.05), but that had higher concentration than donkey milk (*p* < 0.05). Among the anteiso-BCFA, the anteisoC15:0 and anteisoC17:0 showed the highest concentration in sheep milk. The anteisoC15:0 and anteisoC17:0 displayed similar concentration in milk of ruminants (0.46 and 0.42 g/100g of total FA, respectively), whereases in donkey and human samples anteisoC17:0 was about double than anteisoC15:0. Formula milk exhibited the lowest concentration of BCFA, being about 7% to that found human milk (0.04 and 0.55 g/100 g of total FAME, respectively).

Among anteiso-BCFA, anteisoC15:0 and anteisoC17:0 were the most abundant in the milk of all species, except for donkey, that had relatively higher concentration of anteisoC13:0. Among ruminants, anteisoC15:0 was higher in sheep and buffalo compared to the other milks (*p* < 0.05), with cow milk having higher concentration than goats milk (*p* < 0.05); anteisoC17:0 was higher in sheep compared to the other milks (*p* < 0.05), with cow milk having higher concentration than buffalo and goats milks (*p* < 0.05), which did not differ among them. Donkey, human, and formula milks had lower concentrations of anteisoC15:0 and anteisoC17:0 than ruminant milks (*p* < 0.05).

Table 2 shows the percentage of individual OBCFA and the sums of linear-OCFA and BCFA on the total OBCFA. Milk from monogastric species and the formula substitutes showed a greater proportion of linear-OCFA than BCFA. Of the total content of OBCFA, the linear-OCFA was 68.7% in donkey and 59% in human milk, whereas they represent about the 80% of the total OBCFA in formula (Table 2). The content of linear-OCFA in ruminant’s milk ranged from 44.5 to 51.9% of total OBCFA.

### 3.2. Principal Component Analysis

**Figure 1:**
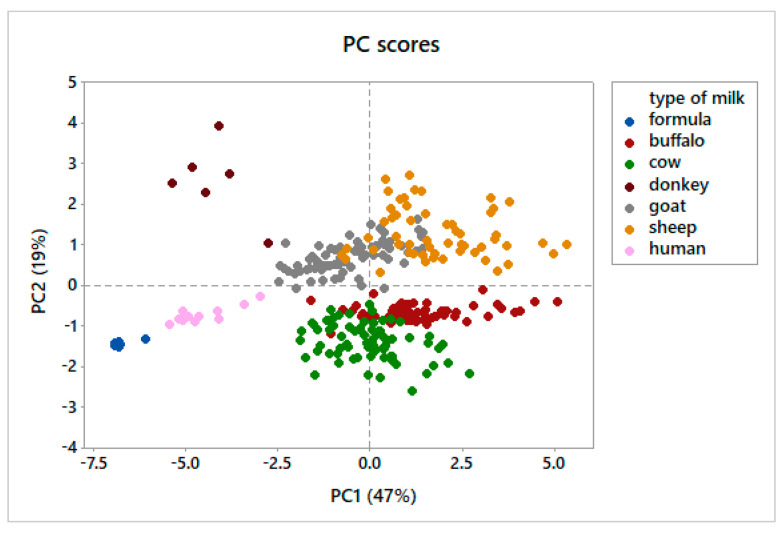
Score plots of the two first principal components explaining 47 and 19% of the total variance, respectively.

About the 88% of the total variance was explained by the first four principal components (PCs), with the two first PCs accounting for about the 70% (PC1 and PC2, 47% and 19%, respectively). Figure 1, reporting the plot of the scores of the PC1 and PC2, allows to identify some clusters of the different types of milk.

Sheep and buffalo milks have generally positive scores for PC1, whereas donkey, human and formula milks have negative scores. Cow and goat milks had mean scores for PC1 close to zero, with a large range of values. For the PC2, milks of donkey, goat, and sheep had positive scores, while those of buffalo, cow, human, and formula milks had negative scores.

**Figure 2:**
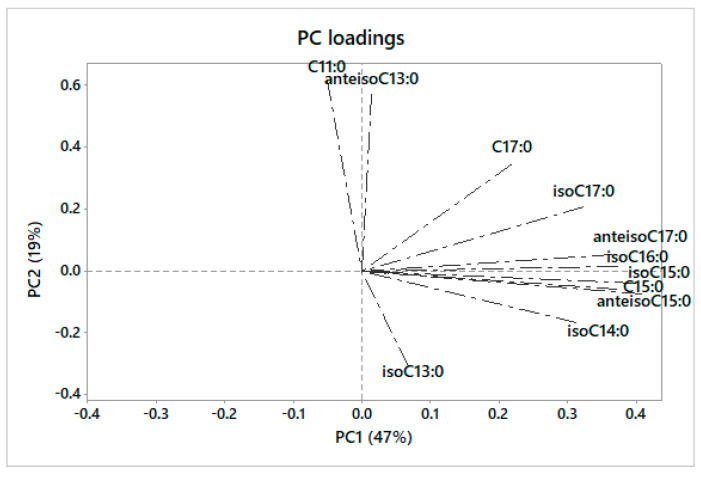
Loadings plot of the two first principal components explaining 47 and 19% of the total variance, respectively.

Figure 2 shows the plot of the PC loadings that can be useful to describe and explain the main differences in term of OBCFA among the clusters of milk observed in Figure 1.

The PC1 had high positive loadings for C15:0, isoC15:0, isoC16:0, anteisoC15:0 and anteisoC17:0. The PC2 had high positive scores for C11:0 and anteisoC13:0, whereases it had high negative scores for isoC13:0.

## 4. Discussion

### 4.1. Univariate Analysis

The mean fat contents observed in ruminants, donkey, and formula milks, were in agreement with previous works [36,37,38]. The fat content of human milk was lower compared to the mean reported in the literature [39]. It should be highlighted that, for the present work, human milk was not sampled from the total daily milk production, but it was spot sampled. Despite this, the fatty acid composition can be considered representative of whole milk production, according to previous observations [40].

The generally higher concentration of OBCFA found in ruminants compared with non-ruminants was expected and in agreement with previous studies [7,41]; it is mainly related to the presence of ruminal bacterial population that synthesize de novo these FA, that are incorporated in their cell membranes. For that, OBCFA can give information about the nutritional status of animals, because the unbalance of some nutrients could lead to a variation of the microbial population, and consequently of these FA. In ruminants, the milk linear-OCFA are considered not exclusively of rumen microbial origin; in particular, a higher content of C17:0 in milk than that found in duodenal flow, suggests a partial synthesis of this FA in the mammary gland [10,26]. This double origin could partially explain the higher concentration of these FA in ruminant milk compared to non-ruminant milk [42].

The concentration of OBCFA found in sheep and goat milk was similar to that reported in previous studies [43,44]. The concentration of these FA in cow and buffalo was lower than that measured by other authors [45,46]. The higher concentration of OBCFA found in sheep milk compared to cow, goat, and buffalo milks could be associated with the different feeding management of these species. The same factor can partially explain differences among works conducted within the same species. For example, OBCFA concentration could be affected by the forage to concentrate ratio [6,10], by the presence of bioactive compounds in the diets [11,12] and by the energy balance of animals [5]. Among monogastrics, the donkey milk had higher amounts of OBCFA than human milk, partly because of the low ability of human tissues to produce OBCFA [9], but mainly because the donkey, being herbivorous, is characterized by the abundant activity of intestinal microflora for fiber digestion [47]. In fact, monogastric herbivores are characterized by a high fermentation activity in the cecum that is responsible for the production of these FA. Regarding this species, the relatively high proportions of C11:0 and isoC13:0 found in the present work (representing the 40 and 6.5% of the total OBCFA respectively) was in contrast with a previous work, where the most abundant OBCFA were C15:0 and C17:0, similarly to what was observed for the other considered species [7]. However, it should be highlighted that different donkey breeds (Sarda breed, Italy) were involved in our and the previous work (Chinese breed).

Products from ruminants are the main source of linear-OCFA in the human diet [48]. However, recent studies showed that C15:0 and C17:0 have different origins in human tissues [15,25]. Indeed, the concentration of C15:0 is strongly correlated to dietary intake, whereas for C17:0 the relationship is weaker [49]. Jenkins et al. [25] highlighted that part of C17:0 is produced endogenously through the elongation of propionyl-CoA and α-oxidation of C18:0. For this reason, C15:0 is a better biomarker of dairy products intake, rather than C17:0. Moreover, the higher concentration of C17:0 found in the human milk compared to the other OBCFA could be due, in part, to its intermediate chain length that can easily fit into lipid structures of triglycerides, phospholipids, and glycosphingolipids [50]. Due to the strong association between C15:0 and the reduction of several diseases (such as reduction of proinflammatory, profibrotic states in human cells and anemia) and the strong correlation between the circulation of this FA in human blood and its intake, the consumption of dairy products might be considered as a strategy able to increase this FA and, consequently, human health.

The high content of BCFA found in sheep could be also associated to a different de novo synthesis of these FA in mammary glands. For example, methylmalonyl-CoA is better used from sheep compared to cow and goat [10]. Methylmalonyl-CoA is the carboxylation product of propionate that could be incorporated at the first step of chain elongation, which might result in the production of anteiso-FA.

In ruminants the iso- and anteiso-BCFA origins are also different, as the iso form arises from isobutyrate and isovalerate, while the anteiso form originates from 2-methybutyrate. The proportion of iso and anteiso is affected by several factors, such as the availability of the precursors and the amount of chain extender, which are related to microorganism activity [51]. For this reason, a change of the ratio odd-chain iso- to anteiso- chain fatty acids could give information about rumen function. A reduction of this ratio could be related to an increase of concentrate levels in animal diets that could lead to a decrease of pH and a consequent change in the rumen bacterial population [10]. Thus, a higher proportion of concentrate in the diet may explain the higher iso to anteiso ratio observed in this work for buffalo milk compared to the other ruminant milks.

Even if the main amount of BCFA arises from rumen bacteria, Vlaeminck et al. [52] showed that an endogenous synthesis of them should not been excluded. Results from Gómez-Cortés et al. [53] supported the hypothesis that a fraction of iso and anteiso C17:0 derived from the endogenous synthesis through the elongation of isoC15:0 and anteisoC15:0. Regarding the concentration of BCFA on human milk, little information is available due to their lower concentration. Generally, the content of BCFA in human milk is associated with the intake of dairy products, even if a small amount could be synthetized in the maternal intestinal bacteria and then transported into milk [9].

Regarding the formula, the concentration of OBCFA is markedly lower compared to the other milks (5% compared to cow milk). This is because there are no sources or poor sources of these FA in the fat of formula milks, being mostly based on vegetable oils [54]. The lower content of OBCFA in these products compared to human milk (10%) needs to be carefully considered, because of the use in infants’ nutrition. The OBCFA in human milk play an important role in the nutrition of newborns and for the neonatal gastrointestinal tract [55]. Moreover, these FA have a protective effect against the necrotizing enterocolitis that could affect premature infants and, for this reason, the presence of OBCFA is necessary also in formula milks [16].

Donkey milk is considered another substitute for human milk, in particular for infants or people with an intolerance for cow’s milk. In terms of OBCFA content, this milk had a higher percentage compared to human milk. However, this milk suffers for a very low fat content. In the present work, the mean was 0.4%, in agreement with previous studies on indigenous Italian, Greek, and Cypriot donkey breeds [56,57]. Due to the low fat content, in children below six months, whose diet is exclusively based on donkey’s milk, the fat levels require a supplementation, e.g., by adding vegetable oils [58].

### 4.2. Principal Component Analysis

The principal component analysis was conducted in order to highlight the overall differences among the different types of milk considered in the present study, and to find possible clusters among them, based on the odd and branched fatty acid composition.

This statistical multivariate analysis is considered a useful approach to reduce the number of the original variables, such as FA, and to reassume the main variability of the system in few new variables (i.e., principal components, PCs). This technique has different applications, including that related to the ability to separate samples (e.g., milk, dairy products or meat of different species, breed, dietary treatment, season of production, etc.), based exclusively on the FA composition [59,60,61].

From the plot of the scores of the two first PCs some considerations can be tried: PC1 seems to separate milks of different species, based on their usual milk fat content. Moving from positive to negative scores, sheep and goat form the first cluster, followed by cow and goat and then by human and donkey; even if formula milks had a mean fat content of 3% (similar to cow milk), these products formed a unique cluster with negative scores, probably due to the vegetable origin of this fat. Indeed, vegetable oils are typically used, (in partial or total substitution of milk fat) to provide the essential fatty acids in infant formula [54].

Looking at the PC2, a certain separation can be observed between small and large ruminants; the same PC provides evidence for the positive scores of donkeys, similar to small ruminants, and negative scores of human and formula milk, more like large ruminants; negative scores for human milk may be related to the human consumption of cow milk or dairy products; negative scores for formula milks can be related to the use of cow’s milk and derivates for the formulation of these products. Looking at the PC loadings, the separation between small and large ruminants is mainly related to differences in the concentration of isoC13:0, isoC14:0, C11:0, and anteisoC13:0 in these species. As previously reported, differences in the OBCFA composition can be related to several factors, in particular, differences of synthesis of these classes of microbial FA may be related to different feeding, BCFA being mainly produced by cellulolytic bacteria, and linear-OCFA by amylolytic bacteria [62]; differences in terms of carbohydrate fermentation and passage rate also cannot be excluded.

Overall, considering both the PCs, donkey milk forms a unique cluster, far from the other milks; considering the PC loadings (Figure 2), this unique cluster is related to a very high proportion of C11:0 and anteisoC13:0 compared to the other milks. These two FA can be then considered as markers, useful to identify donkey milk and derived products.

According to our findings, in a recent work, human milk and donkey milk formed two different clusters, well differentiated from the clusters including other milks, even if in that work all the FA were taken into account [7].

Interestingly, the cluster of formula milks (Figure 1) is quite different from the others: an overlapping of all the samples (except for one) can be observed, suggesting a huge standardization of these milks, even if produced by different companies.

## 5. Conclusions

In conclusion, milk from ruminants and monogastric animals and infant formula strongly differ from each other in terms of OBCFA. Sheep, goat, cow and buffalo milks have higher concentration of these FA compared to donkey, human and formula milks. The differences found among the species depend mainly on the different origins of them. Human milk has moderate concentration of OBCFA and part of them arise from the diet and, in particular, from the intake of dairy products. Considering the recent observations on the beneficial aspects of OBCFA on human health, the lowest concertation of these FA found in formula draws the attention to improve the nutritional value of some substitutes of breast milk.

## Figures and Tables

**Table 1:** Individual and groups of odd- and branched-chain fatty acids (g/100 g of FAME) of formula milk and milk from buffalo, cow, donkey, goat, sheep, and human.

	Cow		Buffalo		Sheep		Goat		Donkey		Human		Formula Milk	
Item	Mean	SD	Mean	SD	Mean	SD	Mean	SD	Mean	SD	Mean	SD	Mean	SD
Fat content, %	3.746 ^d^	0.621	7.581 ^a^	1.421	6.050 ^b^	1.386	5.415 ^bc^	3.070	0.390 ^e^	0.207	1.076 ^e^	0.708	3.004 ^cde^	1.210
*Individual FA*														
C11:0	0.085 ^d^	0.039	0.024 ^e^	0.014	0.286 ^b^	0.088	0.186 ^c^	0.042	0.895 ^a^	0.398	0.010 ^de^	0.009	0.004 ^de^	0.006
C15:0	1.063 ^b^	0.273	1.153 ^ab^	0.176	1.178 ^a^	0.171	0.751 ^c^	0.168	0.374 ^d^	0.085	0.375 ^d^	0.101	0.056 ^e^	0.043
C17:0	0.455 ^c^	0.105	0.506 ^b^	0.076	0.760 ^a^	0.104	0.791 ^a^	0.079	0.353 ^d^	0.109	0.407 ^cd^	0.080	0.078 ^e^	0.025
isoC13:0	0.091 ^a^	0.047	0.020 ^bc^	0.009	0.026 ^b^	0.010	0.025 ^b^	0.005	0.026 ^bc^	0.017	nd	-	nd	-
isoC14:0	0.120 ^b^	0.055	0.189 ^a^	0.04	0.118 ^bc^	0.033	0.099 ^cd^	0.031	0.063 ^de^	0.041	0.020 ^ef^	0.009	0.002 ^f^	0.005
isoC15:0	0.218 ^b^	0.052	0.318 ^a^	0.058	0.293 ^a^	0.079	0.185 ^c^	0.042	0.087 ^d^	0.039	0.054 ^d^	0.029	0.008 ^d^	0.015
isoC16:0	0.233 ^c^	0.070	0.391 ^a^	0.086	0.332 ^b^	0.059	0.245 ^c^	0.083	0.140 ^d^	0.041	0.082 ^de^	0.031	0.007 ^e^	0.015
isoC17:0	0.253 ^c^	0.066	0.241 ^c^	0.042	0.415 ^a^	0.079	0.311 ^b^	0.094	0.058 ^de^	0.035	0.127 ^d^	0.055	0.006 ^e^	0.013
AnteisoC13:0	nd	-	0.036 ^b^	0.007	0.043 ^b^	0.013	0.022 ^c^	0.007	0.154 ^a^	0.121	nd	-	nd	-
AnteisoC15:0	0.444 ^b^	0.106	0.540 ^a^	0.096	0.562 ^a^	0.127	0.329 ^c^	0.060	0.072 ^d^	0.031	0.086 ^d^	0.040	0.011 ^d^	0.019
AnteisoC17:0	0.415 ^b^	0.102	0.365 ^c^	0.067	0.500 ^a^	0.086	0.388 ^bc^	0.078	0.138 ^d^	0.055	0.181 ^d^	0.048	0.005 ^e^	0.012
*Groups of FA ^1^*														
linear-OCFA	1.603 ^b^	0.362	1.683 ^b^	0.222	2.223 ^a^	0.282	1.729 ^b^	0.247	1.621 ^b^	0.331	0.792 ^c^	0.173	0.138 ^d^	0.069
iso-BCFA	0.916 ^b^	0.163	1.159 ^a^	0.203	1.185 ^a^	0.228	0.865 ^b^	0.190	0.374 ^c^	0.148	0.283 ^cd^	0.119	0.022 ^d^	0.037
anteiso-BCFA	0.858 ^b^	0.185	0.941 ^b^	0.159	1.105 ^a^	0.196	0.739 ^c^	0.118	0.363 ^d^	0.095	0.267 ^d^	0.086	0.016 ^e^	0.031
BCFA	1.775 ^c^	0.303	2.100 ^b^	0.356	2.290 ^a^	0.397	1.604 ^d^	0.286	0.738 ^e^	0.185	0.550 ^e^	0.202	0.038 ^f^	0.066
OBCFA	3.378 ^c^	0.494	3.783 ^b^	0.538	4.513 ^a^	0.577	3.333 ^c^	0.499	2.359 ^d^	0.424	1.342 ^e^	0.364	0.176 ^f^	0.133

^1^ linear-OCFA = odd-chain fatty acids (sum of all individual linear odd-chain FA); iso-BCFA = iso branched-chain fatty acids (sum of all individual iso-branched chain FA); anteiso-BCFA = anteiso branched-chain fatty acids (sum of all individual anteiso-branched-chain FA); BCFA = branched-chain FA (sum of all branched-chain FA); OBCFA = odd- and branched-chain FA (sum of all odd- and branched-chain FA). Different superscripts in the same row indicate significant difference (*p* < 0.001) among type of milk. n.d. indicates not detected FA. SD indicates standard deviation.

**Table 2:** Percentage of individual odd and branched fatty acids of the total of OBCFA considered.

	Type of Milk						
	Cow	Buffalo	Sheep	Goat	Donkey	Human	Formula
*Individual FA*							
C11:0	2.5	0.6	6.3	5.6	37.9	0.7	2.3
C15:0	31.5	30.5	26.1	22.5	15.8	27.9	31.8
C17:0	13.5	13.4	16.8	23.7	15.0	30.3	44.4
isoC13:0	2.7	0.5	0.6	0.8	1.1	nd	nd
isoC14:0	3.6	5.0	2.6	3.0	2.7	1.5	1.0
isoC15:0	6.5	8.4	6.5	5.5	3.7	4.0	4.3
isoC16:0	6.9	10.3	7.4	7.4	5.9	6.1	3.8
isoC17:0	7.5	6.4	9.2	9.3	2.5	9.4	3.4
anteisoC13:0	nd	0.9	0.9	0.7	6.5	nd	nd
anteisoC15:0	13.1	14.3	12.5	9.9	3.0	6.4	6.0
anteisoC17:0	12.3	9.6	11.1	11.6	5.8	13.5	3.1
*Groups of FA ^1^*							
linear-OCFA	47.5	44.5	49.3	51.9	68.7	59.0	78.5
BCFA	52.5	55.5	50.7	48.1	31.3	41.0	21.5

^1^ linear-OCFA = linear odd-chain fatty acids (sum of all individual linear odd-chain FA); BCFA = branched-chain FA (sum of all branched-chain FA).

## Data Availability

Data are contained within the article.
